# Supplementary material for: Are carbon emissions trading and green financial instruments synergistic? -Comprehensive quantitative research based on content analysis
Source: PLoS One. 2024 Mar 7;19(3):e0298601. doi: 10.1371/journal.pone.0298601 (PMC10919601; doi:10.1371/journal.pone.0298601)
Supplement: S1 Data — (ZIP) [file pone.0298601.s001.zip › Data/地级市Z_F工作报告.zip!/╜¡╬≈/╛┼╜¡╩╨/└┤╫╘╣1⁄2╓┌║┼í╛┬φ┐╦╩2╛▌═°í┐_2012.docx]

各位代表：

　　现在，我代表市人民政府，向大会作政府工作报告，请予审议，并请各位市政协委员和列席会议的同志提出意见。

　　一、2011年工作回顾

　　过去的一年，面对复杂的宏观经济环境和春夏连旱、旱涝急转等自然灾害的严峻考验，在省委、省政府和市委的正确领导下，全市上下坚持以科学发展观为指导，以鄱阳湖生态经济区建设为龙头，紧紧围绕“两区互动、强工兴城”工作重点，不因快速发展而自满，不因换届而分神，不因遇到困难而退缩，乘势而上，全力赶超，实现了“十二五”的良好开局。

　　（一）财政收入三年翻番目标超额完成，经济总量进一步扩张。全市实现生产总值1256.4亿元，同比增长13.2%。财政总收入161.8亿元，增长38.6%，超额完成市委九届七次全会确定的“财政收入三年翻番”目标；财政总收入占GDP比重提高1.6个百分点，达到12.9%，其中地方一般预算收入101亿元，增长42.2%；县级财政收入135.1亿元，占全市83.5%；县县财政收入实现“三年翻番”，由2008年一县过5亿到县县过5亿、六个过10亿，九江经济技术开发区突破15亿，实现“一年翻一番”。全市社会消费品零售总额329.7亿元，增长18.1%。年末全市金融机构本外币各项存款余额1255亿元，增长15.4%；贷款余额804亿元，增长24.1%，增幅全省第一。完成进出口总额37.9亿美元，增长109%，其中出口总额26.3亿美元，三年增长9倍，增幅全省第一，总量首次位居全省第二；引进省外5000万元以上项目150个，实际进资307.1亿元，实际利用外资7.7亿美元，开放型经济工作综合考评连续三年全省第一。民营企业户数2.3万户，非公有制经济在全市经济中“三分天下有其二”。

　　（二）决战工业2000亿目标提前一年实现，工业实力进一步壮大。全市规模以上工业主营业务收入2189.1亿元，增长50.8%，总量两年翻一番，“三年决战工业2000亿”目标两年完成，工业化率提高2.8个百分点；规模以上工业增加值481亿元，增长18.3%，净增额首次突破100亿元；利税总额200.9亿元，增长33.3%。通过龙头引领、延链配套，引导企业抱团扎堆，主导产业加速集聚。十大产业实现主营业务收入1968.1亿元，增长54%，占全市比重89.9%。通过政策扶持、调度推进，鼓励企业技改扩能，骨干企业加速壮大。主营业务收入过亿元企业496户，净增176户；过10亿元企业25户，净增8户；九江石化、九江钢厂突破200亿元。通过加大投入、加强配套，工业平台加速拓展，承载能力不断增强。完成园区基础设施投入108.8亿元，增长54%，投入总量和增幅均列全省第一。全市园区主营业务收入1728.7亿元，过百亿园区7个，净增3个，九江经济技术开发区实现371亿元。

　　（三）决战城建200亿目标提前一年实现，城市形象进一步提升。中心城区实施城建重点项目135个，完工61个，全年完成投资近200亿元，两年投入近300亿元，“三年决战城建投入200亿”任务提前一年完成，全市城镇化率提高1.86个百分点。大九江格局加快构筑。105国道（环庐山段）等区间通道和九码快速通道建成通车，九江县对接八里湖新区“两纵两横”路网等项目快速推进，大九江20分钟城市圈交通框架基本成型。各县（市、区）共实施项目872个，总投资350多亿元。3个省级示范镇、11个市级示范镇建设进展顺利。八里湖新区功能加快完善。完成60平方公里控制性详规修编。实施城建项目60个，完成政府性投资30亿元，带动社会投资100亿元。市第一人民医院总院、九江一中新校区、新体校、城市展示馆等项目投入使用，八里湖大桥、东组团路网和地下管网工程基本完工，市民服务中心、文化艺术中心、博物馆、蛟滩水利综合整治工程等项目加快推进，新区市政基础、公共服务、生态环境、商住配套等设施逐步完善。老城区改造加快推进。浔阳路、浔阳西路、十里大道、大中路西段历史文化街区改造全面完成，桃园路、欣荣路、浔阳东路地下人防工程等项目投入使用，南山广场及公园等项目基本建成，滨江西大道、环湖北路、赛城湖公园周边环境整治等项目顺利推进。老城区功能不断完善，人居环境不断优化。城市管理不断加强。以交通秩序、市容环境、建筑工地等为重点的城市管理专项整治行动成效明显，“两声三乱”整治成果得到巩固。大九江范围内“两违”整治强力推进，拆除各类违法违规建筑1500户近20万平方米。“六城同创”稳步推进，六次蝉联全省双拥模范城,成功创建全国无障碍建设先进城市，顺利通过国家园林城市评审和省级卫生城市复审。

　　（四）固定资产投资突破1000亿元，发展后劲进一步增强。坚持重大项目带动战略，实施亿元以上重大项目376个，其中10亿元以上22个。500万元以上的固定资产投资1006.5亿元，增长39.4%，总量全省第二，增幅全省第一。一批重大产业项目快速推进。投资80亿元的星火有机硅一体化、80亿元的旭阳雷迪三期、50亿元的江铜铅锌冶炼、50亿元的攀森多元金属镍粒、30亿元的龙达粘胶纤维、30亿元的中粮植物油、30亿元的赛翡蓝宝石、25亿元的九钢技改等项目投产，投资100亿元的九江石化油品质量升级、100亿元的理文造纸、100亿元的恒生大化纤、50亿元的天然气储备及利用工程、45亿元的九电四期、26亿元的亚泥五六期等项目开工建设。一批重点基础设施项目快速推进。永武高速、景湖公路建成通车，九江长江公路大桥、彭湖沿江大道、修万公路、定龙公路等项目进展顺利。老爷庙风电并网发电，全市扩建220千伏变电站2个，新建110千伏变电站4个，新增主变容量50万千伏安，新建线路171公里。一批重要社会事业项目快速推进。市美术馆、科技中专新校区、特教学校康复楼等项目交付使用，九江学院附属医院新院区、双峰小学分校等项目进展顺利。

　　（五）“三农”工作扎实推进，农村生产生活条件进一步改善。农业产业化加快推进。大宗农产品稳产增产，粮食产量实现“八连增”。全年落实惠农补贴2.8亿元，农机购置补贴4264万元。建立省级以上无公害、标准化产业基地57个，全市农村土地流转面积80万亩，新增农民专业合作社360家。新增规模以上龙头企业20家，总数达322家，实现主营业务收入404亿元。新增省著名商标22个、省名牌5个，农产品市场竞争力不断提高。基层农技推广体系建设继续走在全国前列，新增全国示范县2个。基础设施建设大力推进。争取上级水利资金13亿元，共投资18亿元，完成病险水库除险加固、中小河流治理、农村安全饮水、小农水重点县等一批水利工程项目。完成各类水利工程3463座（处），新增、恢复、改善灌溉面积26.4万亩，改造中低产田8.2万亩。改渡建桥33座，改造农村公路913公里，新建乡镇客运站42个。新农村建设稳步推进。财政投入8500多万元，启动395个贫困村整村推进，完成扶贫移民搬迁4161人、避灾移民搬迁7236人。整合资金3.2亿元，以村庄整治为切入点，建设新农村建设点1139个，开展农村清洁工程试点3268个，新农村建设连续四年保持全省领先。

　　（六）鄱阳湖生态经济区战略深入实施，发展方式进一步转变。生态建设不断加强。造林绿化47.6万亩，治理水土流失24万亩。林权制度配套改革深入推进，全市林权交易面积24.6万亩。新增省级生态镇12个、生态村15个。积极开展环保专项行动，重点耗能企业和公共机关节能有效加强。大气环境优良率达99.4%，主要河流断面水质达标率100%，省政府下达的节能减排任务全面完成。新兴产业不断发展。新能源、非金属新材料、电子信息、节能电器、绿色食品五大新兴产业实现主营业务收入730亿元，占全市比重超过三分之一。全市共有9个工业园区申报省级生态园区建设，3个通过验收。第三产业不断成长。旅游经济主要指标继续保持全省前列，全年接待游客2868.8万人次，旅游总收入205.7亿元。金融、保险、证券业加快发展，城投、国资、置地、八里湖等平台的融资水平明显提高，有效保障了改革、建设和创业的资金需求。港口集装箱吞吐量超过14万标箱，开通了九江港至韩国仁川港、上海洋山港始发直达货运班轮。建设标准化农家店225家、商品配送中心6个，“家电下乡”销售总额15.3亿元。九江粮食现代物流中心投入运营，九江现代综合大市场、九江国际汽车城改扩建、大润发广场等商贸物流项目稳步推进。

　　（七）社会事业协调发展，人民生活水平进一步提高。城镇居民人均可支配收入17911元，增长13.6%；农村居民人均纯收入6778元，增长21.3%。民生工程扎实推进。整合资金35.2亿元用于保障和改善民生，省政府下达的民生工程任务全面完成，市定的民生实事基本完成。投资24.7亿元建设保障性住房3万套；投资25.8亿元改造棚户区256.4万平方米，近2万户居民居住条件得到改善；发放租赁补贴1610万元，惠及7404户，保障性住房建设工作走在前列，全省兴起“外学重庆，内学九江”的热潮。新增城镇就业5.8万人，新增转移农村劳动力近7万人，发放小额贷款6.3亿元，城镇就业率98％，“零就业”家庭实现动态清零。城乡低保补助标准逐步提高，“五保”集中供养率 85%，医疗救助37.1万人次，城镇基本养老保险参保47.3万人，被征地农民养老保险工作有效推进；城镇居民基本医疗保险参保82.4万人，在校大学生全部纳入保险范围；新型农村社会养老保险试点覆盖面71.3％，新型农村合作医疗参合率98.5%。免费开展白内障复明手术18701例、唇腭裂修补手术899例；城乡困难群众免费体检、尿毒症患者免费血透和儿童白血病、先天性心脏病免费治疗等医疗救助工作扎实推进。新建改造城区农贸市场5个，扩建城郊蔬菜基地6000亩。切实加强价格监管，居民消费价格指数105.1%，低于全国全省平均水平。更新市区公交车86辆，市民出行更便利。大力实施质量兴市战略，切实加强质量监管，严厉打击制售假冒伪劣商品违法行为，“塑化剂”、“瘦肉精”、“地沟油”等食品安全专项整治成效明显。社会管理不断加强。民声直通车共受理群众诉求11853件，直通房产、医疗、教育等专题活动社会效果良好。落实信访责任制和信访接待日制度，信访办结率、停访息诉率、群众满意率不断提高。强化安全生产监管，安全事故发生率和安全事故死亡人数双下降。加强治安防控体系建设，依法打击各类违法犯罪活动，社会治安持续稳定，公众安全感测评全省第一。社会事业全面发展。争取省部级科技项目79项，获省科技进步奖3项，专利授权450件，获批国家高新技术特色产业基地1个，新增国家高新技术企业8个。各类教育均衡发展，新建改造农村中小学校舍35万平方米，新招农村教师1600人。建成乡镇综合文化站68个、农家书屋550家，申报省级文化产业示范基地6个。农业、林业、水利、交通、商贸、粮食六大非工系统国企改革全面完成，农垦系统改革顺利推进。医药卫生体制改革深入推进，国家基本药物制度实现全覆盖，农村三级卫生服务网络和社区卫生服务体系建设不断加强。第六次人口普查顺利完成，人口自然增长率7.35‰。被评为“全国人口和计划生育综合改革示范市”和“全国全民健身先进单位”。统计、审计、海关、口岸、检验检疫、应急管理、气象、水文、盐业、通讯、无线电管理、信息化、档案、保密、编制、地方志、对台、外事侨务、民族宗教等工作得到新加强。国防动员、民兵预备役、军民融合、人民防空、防震减灾、消防、国家安全等工作取得新成绩。工会、共青团、妇女儿童、老龄、残疾人、红十字和慈善等事业实现新发展。

　　（八）政府自身建设不断加强，政务环境进一步优化。市、县、乡三级政府换届圆满完成，全市上下促赶超的积极性不断高涨，发展动力和活力进一步增强。按照“核心党委、高效政府”的要求，坚决落实市委的决策部署，自觉接受人大法律监督和政协民主监督，认真听取各民主党派、工商联和无党派人士意见，办理市人大代表建议354件、市政协提案746件。强化行政效能监察，“十百千万”工程扎实开展，发展提升年活动成效明显。推进政务公开，规范行政审批事项，清理面向基层“一票否决”事项43项，网上审批事项12.5万件。积极开展政风行风评议，加大对工程建设、土地、民生等领域突出问题专项治理，集中开展民生领域“新三项”问题、医药购销和医疗服务中突出问题、公路“三乱”等专项治理行动，行政权力运行监控机制进一步完善，反腐倡廉惩防体系进一步健全。

　　各位代表，这些成绩的取得，是省委、省政府和市委坚强领导的结果，是市各套班子共识共为的结果，是全市人民团结奋斗的结果。在此，我代表市人民政府，向各位人大代表、政协委员，向奋斗在各条战线的广大干部群众，向离退休老同志，向各民主党派、工商联、无党派人士、各人民团体，向驻浔人民解放军和武警官兵，向中央和省驻浔单位，向所有关心、支持和参与九江建设的海内外朋友，表示衷心的感谢并致以崇高的敬意！

　　在看到成绩的同时，我们也清醒地认识到，经济总量不大、发展不足的市情尚未根本改变，赶超进位的任务仍然艰巨；工业化、城镇化水平不高的局面尚未根本改变，强工兴城的任务仍然艰巨；群众收入水平偏低、部分居民生活还比较困难的现状尚未根本改变，提高收入和改善民生的任务仍然艰巨；县域经济还不够强，壮大县域经济的任务仍然艰巨；影响和谐稳定的因素依然存在，社会建设和管理面临的任务仍然艰巨；一些制约发展的体制、机制难题有待进一步破解，发展环境有待进一步优化，行政效能有待进一步提高。对此，我们将采取有力措施，切实加以解决。

　　二、2012年工作安排

　　2012年是实施“十二五”规划承上启下的重要一年，是实现赶超目标、争得应有地位的攻坚之年。尽管世界经济形势十分复杂严峻，我国经济发展中不平衡、不协调、不可持续的矛盾和问题仍然突出，但国内经济平稳较快发展的基本面没有改变，全省进入新的快速发展阶段，我市仍处于加速赶超的机遇期；尽管区域竞争更加激烈，赶超进位的任务更加艰巨，但我市近年来固投积蓄的巨大能量加速释放，鄱阳湖生态经济区战略的政策效应逐步显现，省委、省政府将沿江开放开发定位为推动区域发展的新引擎，以此昂起鄱阳湖生态经济区产业经济发展的龙头，九江的资源和区位优势加快放大，我市将迎来加速赶超的爆发期；尽管前进中将面临困难和挑战，但在市委的坚强领导下，各级班子活力和干劲进一步增强，各级干部操作力和执行力进一步提高，赶超发展的氛围更加浓厚，合力更加强大，我市已进入加速赶超的黄金期。我们既要科学应对面临的挑战，更要抓住用好难得的机遇，进一步坚定赶超发展的信心，顺势而为，乘势而上。

　　今年政府工作的总体要求是：坚持以邓小平理论和“三个代表”重要思想为指导，深入贯彻落实科学发展观，全面贯彻省第十三次、市第十次党代会精神，围绕“九江争得应有地位、人民过上小康生活”的奋斗目标，推进鄱阳湖生态经济区建设，突出发挥沿江优势，决战大工业，建设大九江，坚持攻沿江、抓项目、扩投资、惠民生，全力加快赶超步伐，以优异的成绩迎接党的十八大胜利召开。

　　全市经济社会发展主要预期目标是：生产总值增长13%；全社会固定资产投资增长25%；财政总收入增长18%以上；社会消费品零售总额增长18%；居民消费价格涨幅控制在4%左右；城镇居民人均可支配收入和农村居民人均纯收入增长12%；人口自然增长率控制在8‰以内；完成省政府下达的节能减排任务。

　　为实现上述目标，主要抓好八个方面的工作：

　　（一）以决战沿江大工业为重点，推动工业经济再跃新台阶

　　按照省委、省政府“加快打造由南昌核心增长极、九江沿江产业带、昌九工业走廊构成的核心增长区”的决策部署，充分利用152公里长江岸线资源，全力决战大沿江，联动昌九大走廊，昂起鄱阳湖生态经济区产业经济发展的龙头，打造带动区域发展的新引擎。

　　举全市之力加快沿江大开发。坚持规划引领，实施高起点开发。按照“一港三区”的目标，加快完善沿江开放开发总体规划，重点做好城西、城东、赤码、彭湖四大板块产业发展规划，科学修编港口码头、土地利用、交通基础设施、电网等专项规划，着力打造长江中下游区域重要交通枢纽港、沿江产业区、现代物流区、城镇组群区。坚持基础先行，实施建设性开发。大力推进沿江“十大基础设施工程”建设，加快启动彭湖、赤码两大板块的公共物流码头和城西港区、湖口港区铁路专用线建设。全力支持上港集团做大做强，推进南昌港区、彭湖港区、赤码港区联动发展。加大沿江通道和输变配电网建设力度，年内建成彭湖沿江大道、赤码沿江大道主体工程，建成九化、彭泽红光、瑞昌码头等变电站。坚持项目带动，实施产业型开发。把重大项目作为沿江开放开发的主抓手，加快推进在建大项目，强化招商承接新项目，包装策划吸引大项目，盘整清理提升老项目。从投资总量、投资强度、产业类别、产业规模、税收贡献等方面制订产业项目准入标准，充分放大沿江岸线和沿江区域未利用地的优势，加快引进龙头带动型、延链配套型、同类扎堆型等项目向沿江聚集。大力发展石油化工、钢铁和有色冶金、装备制造、新材料、电力能源和化纤纺织等产业，着力推进沿江“十大产业工程”项目建设，促进临港产业集群式发展，力争年内沿江新增1-2户主营业务收入过百亿元的企业。坚持政策扶持，实施引导式开发。设立3000万元沿江开放开发专项资金，加强沿江开放开发投融资平台建设，进一步拓宽融资渠道，强化资金保障。抓好国家和省相关扶持政策的对接，争取在重大项目布局、项目审批、环境容量、用地、用电、资金等方面的倾斜，积极申报省级彭湖开发区、赤码开发区和九江保税港区。按照“项目共建、利益共享”的原则，从财税、金融、人才、资源等方面加大支持引导力度，鼓励区域外重大项目落户沿江，大力发展临港“飞地经济”。坚持科学高效，实施集约式开发。建立岸线利用约束、管理和评估机制，以标准化、公共性码头建设为主，集中开展岸线、码头专项整治，探索企业自用码头开放机制，提高岸线资源利用效率。加强沿江项目的盘整清理，重点向“三高三低”项目倾斜，对不符合产业布局的企业实行“腾笼换鸟”。强化产业布局规划约束，建立项目投资审批、土地供应、环境保护、供电供水等联动机制，严格执行沿江板块工业项目准入标准，真正让黄金岸线产出黄金效益。坚持纵深推进，实施联动式开发。按照“四个相向发展”的格局，以沿江的赤码、城西、城东、彭湖“四板块”，沿路的永修、共青城、德安、沙河、开发区“五组团”为依托，推进沿江板块与沿路板块的协调互动，推进临港产业和沿路产业的配套联动，推进沿江沿路对腹地纵深的辐射带动，打造“T”型工业走廊，使之成为九江发展的核心增长极。

　　举全市之力推动总量大扩张。围绕决战工业6000亿目标，快速扩张工业总量，全市规模以上工业实现主营业务收入3000亿，增长40%以上；增加值580亿元，增长18 %以上。招商引资扩总量。全面加大招商引资力度，以长珠闽和港澳台为主攻方向，以中央和省属企业为主攻重点，有针对性地开展产业招商，大力引进产业链条长、税收贡献大的企业。完善招商目标责任制，落实产业招商专项经费，建立产业招商利益分享机制及调度督查机制，采取专业招商、以商招商、委托招商等多种形式，切实提高招商成功率和产业关联度。力争全年实际利用外资8亿美元，引进内资350亿元。做大企业扩总量。以重点产业项目达产达标为基础，着力打造一批主导市域发展的百亿级航母型企业、支撑县域发展的50亿级旗舰型企业和带动园区发展的10亿级龙头型企业。力促九江石化原油加工量新增100万吨以上，主营业务收入达300亿元以上；力促旭阳雷迪一、二期达产达标，三期全面投产，主营业务收入100亿元以上；力促赛龙通讯扩张产能，主营业务收入80亿元以上；力促昌河汽车扩大整车与发动机产能，主营业务收入50亿元以上，早日实现“3231”目标；力促江铜铅锌冶炼、攀森多元金属镍粒、赛得利粘胶、绿晶光电、铨讯数码等企业主营业务收入30亿元以上；力促超日光伏、赛翡蓝宝石、正展光电、志高空调等企业主营业务收入10亿元以上。集聚产业扩总量。紧紧围绕十大主导产业，主攻一批关联度大、带动力强的重大产业项目，重点培育石化、钢铁、有色冶金、纺织服装、汽车船舶、电子信息、新能源、非金属新材料、节能电器、绿色食品十大产业集群，形成一批新的产业龙头，力争十大产业所占比重提高2-3个百分点。

　　举全市之力服务工业大发展。把做大做强工业作为推动赶超的重中之重，营造服务大工业的浓厚氛围，凝成决战大工业的强大合力。大力帮扶实体经济。加大政府投资、向上争资、市场融资力度，进一步激活民间投资，拓展投资空间，引导市场要素向实体经济聚集。加强财政专项资金扶持，促进企业扩能升级、技术改造、品牌创新。加大金融对企业尤其是中小企业的信贷支持力度，帮扶引导小型微型企业稳健经营、扎堆发展、做大规模，不断增强盈利能力和发展后劲。全面落实支持实体经济发展的优惠政策，切实减轻企业税费负担。扎实推进企业一套表统计制度改革，抓好工业企业名录库、统计培训、企业人力及设备配备、数据衔接等相关工作，增强工业运行质量监测和服务水平。大力服务项目建设。继续实施重大项目审批绿色通道和联审联批制度，不断完善项目建设帮办服务体系，进一步提升项目推进服务水平。全面清理园区闲置用地，坚持依法依规用地、集约节约供地，挖掘用地潜力，提升用地效益，加大以地换地、造地增地、向上争地力度，确保项目用地需求。大力优化园区平台。加强园区水、电、路、气等基础设施建设，加快推进公共服务平台、标准厂房、公租房等生产、生活配套设施建设，增强园区承载力。力争全市工业园区主营业务收入2000亿元以上，百亿园区达9个以上，两个进入全省十强。

　　（二）以八里湖新区开发为重点，推动城市建设再上新水平

　　围绕打造“宜居、宜业、宜游”城市的目标，加速推进八里湖新区开发建设，加快老城区改造提升步伐，统筹推进县域城镇建设，全市城镇化率提高1.5个百分点。

　　着力构建大九江格局。推进“四县一山”与中心城区相向发展、融为一体，逐步形成同城效应。加快“四县一山”与中心城区规划对接，完成大九江城镇群空间发展战略规划和城市综合交通体系规划研究。推进绕城高速建设，完成城市轻轨交通建设规划，逐步建立大九江范围内衔接有序的公共交通体系。本着“规划一步到位，治理分步实施”的原则，围绕市中心城区实现“大水系连通、水环境改善、水安全保障、水生态良好”的目标，启动大水系综合治理工程。提升县城和集镇规划、建设、管理水平，建设一批规划科学、规模适度、设施配套、环境优美、特色彰显的新型城镇。

　　着力打造八里湖新区。按照“大开发、大建设、大提升”的要求，围绕打造“生态新区、特色新区、活力新区”的目标，进一步明确八里湖新区近期建设范围，加快空间走廊建设，拓展新区发展空间。坚持规划引领、项目带动、强力调度、快速推进，建成市民服务中心、市民中心公园、九龙山公园、八里湖大桥、南组团路网等项目；启动市接待中心、八里湖文化长廊、八里湖水面利用及游船开发、文博园、茅山头城市综合体、浔阳古城景区等项目；通过市场引导、政策倾斜等办法，鼓励市直及驻市部门进驻八里湖，加快引进一批有实力、有影响的大企业共同参与八里湖开发建设，大力推进金融中心、中航商业城、中央商务区、特色小镇、国际会展中心等项目建设，促进城市要素向新区集聚，提升新区人气，推动中心城区加快从“两湖时代”走进“八里湖时代”。

　　着力提升老城区品位。大力实施旧城综合改造，着力推进庐山区“整区改造、整体提升”和开发区滨兴片区改造，加快改变旧城整体面貌；大力实施棚户区搬迁改造，按照“分片改造、整体提升”的原则，着力推进浔阳区火柴厂片区、花果园片区、琵琶亭片区改造等工程建设，有效改善棚户区群众居住条件；大力实施市场提升改造，按照“城内大商场、城外大市场”的要求，着力推进烟草物流中心、京九批发市场搬迁等商贸物流项目改造建设步伐，进一步优化布局，提升档次；大力实施环境优化改造，以边街小巷整治为重点，推进环卫设施、群众健身场所等公共服务设施建设，优化人居环境，提升城市品位。

　　着力塑造城市新形象。突出建管并举，按照“条块结合、以块为主”的网格化管理要求，进一步完善“市、区、街、居”共同参与的城市管理格局。突出机制创新，按照“花钱买管理不花钱养人”的理念，制定出台城市公建项目市场化管理办法，积极探索城建项目运营新模式，不断提高城市管理科学化水平。突出集中整治，以“六城同创”为载体，继续推进城市管理专项行动，进一步巩固和扩大“两违”建筑、“两声三乱”、交通秩序、市容市貌、环境卫生整治成果。突出日常监管，大力实施城市清洁工程、畅通工程和美化工程，使城市地面更干净、路面更畅通、立面更亮丽。

　　（三）以增加农民收入为重点，推动“三农”工作再迈新步伐

　　坚持“三农”基础地位不动摇，突出科技兴农、产业富农、基础强农、政策惠农，促进农业增产、农民增收、农村繁荣。

　　完善农业农村基础设施。加强农田水利建设，加快高标准农田建设，抓好水利基础设施管理维护，着力推进“润田工程”、“沃土工程”，完成规划内病险水库除险加固，恢复和新增灌溉面积9万亩，改善灌溉面积18万亩。开展农村信息化试点工作，完善农村通信网络设施，健全农村信息服务体系，建设农村信息服务站300个。继续推进农村电网改造，加快农业机械化步伐，农机总动力增长6%以上。

　　加快农业产业化发展。完善农业科技创新机制，改善农业科技创新条件，构建适应现代农业发展要求的技术体系。加大农业投入和补贴力度，确保播面稳定在400万亩，总产150万吨以上。围绕蔬菜、水产、林果、畜禽、蚕桑、茶叶等特色产业，加快推进产业基地、合作组织、龙头企业、流通市场和农产品加工园区建设。加大农业产业化项目引进力度，力争全年新引进3000万元以上项目70个，支持神州通高产油茶林基地和油茶精深加工项目建设。加快农业产业化龙头企业建设，重点培育80家主营业务收入过亿元的龙头企业，新增规模以上企业20家。引导农业产业化项目向园区集聚，重点建设修水县茧丝绸、永修县果蔬、庐山区油脂等8个农产品加工园区。稳定完善农村土地政策，积极推进土地有序流转，促进农业生产经营模式创新，重点建设好10个万亩、100个千亩以上的产业基地。提升农村金融服务水平，加快农民合作组织、农产品经纪人等流通主体建设，新增农民合作组织300家，逐步健全农业社会化服务体系。加快农业产业化品牌建设，打响“鄱湖水产”等特色品牌，力争创建省级以上农产品品牌30个，无公害、绿色、有机食品品牌50个。

　　扎实推进新农村建设。坚持以自然村建设为主体，以村镇联动和村落连片推进为重点，扎实推进1100个新农村建设点“三清七改五普及” 和“三绿一处理”村庄整治工作。完善农村清洁工程“445”操作模式，完成建设点2000个。强化农民建房管理，引导农民建房向集镇、中心村集中。加强教育科技培训，实施好“阳光工程”、“雨露计划”，完成劳动力转移培训3万人；扎实推进“一村一名大学生”工程，完成633名村级大学生培养计划，造就一批新型农业农村人才。

　　（四）以推进项目大建设为重点，推动赶超发展再添新后劲

　　继续实施项目推进“六个一”机制，强化项目调度，优化项目服务，确保项目早开工、早建成、早投产，力争全社会固定资产投资超过1500亿元。

　　竣工投产一批。加快推进亚泥第5条生产线、昌河汽车、共晶光伏、超日光伏、理文造纸、华光镍铬合金、志高空调二期等产业项目建设，加快修万公路、老九湖路、老九星路改造和蛟滩水利综合整治、三都水电站等基础设施项目建设，力促项目早投产，早见效。

　　续建推进一批。加快推进九江石化油品质量升级、星火有机硅一体化、亚泥第6条生产线、理文化工、恒生大化纤、湖口天然气储备及利用、迅腾硅业、江西中盛陶瓷、九江电厂四期、华电分布式能源、大唐国际永修风电等产业项目建设，加快九江长江公路大桥、105国道九江段改造、都中公路改造、南昌至湖口二级航道整治等基础设施项目，力促项目早竣工，早投产。

　　开工建设一批。加快推进神华煤电一体化、杭氧工业气体、赤湖轻工产业园、德鑫纺织百万精梳二期、青岛啤酒、王老吉饮料、梅兰化工、超高分子聚乙烯、九江逸达造船、厦门钨业硬质合金、华锐风电、维科锂离子电池、南方（彭泽）水泥等工业项目；加快推进九景衢铁路、昌九高速（通远段）改扩建、巾口至南义公路、辽南线和德白公路升级改造、九电四期外送配套、500千伏石钟山至景德镇洪源线路、500千伏马回岭变电工程、220千伏蔡岭输变电等基础设施项目；加快推进九江职业大学、九江职业技术学院和双峰小学等院校新校区，市儿童医院、九江学院附属医院新院区等社会事业项目，力促项目早开工，早竣工。

　　谋划储备一批。力争新增储备亿元以上项目100个，新增储备投资500亿元以上。抓紧做好1000万吨湖口优质钢铁产业基地、大族电池片、永盛聚脂切片、五七二七退城进园、赛得利三期、耐尔电动车、中明特种陶瓷、氯碱产业园、鑫山水泥技改扩能、蒋公岭风电、瑞昌风电、九电五期、沙岭风电、武九客专（瑞昌至九江段）、都昌至九江高速、修水至平江高速、武宁罗溪至靖安石境公路、良山公路改造、市养老中心、九江学院新校区等项目前期工作，力促项目早落地，早开工。

　　（五）以壮大县域经济为重点，推动统筹发展再创新业绩

　　按照“城乡统筹、产业协调”的要求，努力壮大县域经济，积极发展第三产业，促进经济社会全面进步。

　　促进县域经济加快发展。完善激励引导机制、市县联动机制和县域协调机制，鼓励沿江、沿路抢抓机遇，加速做大总量，做强实力，实现跨越发展；鼓励湖区、山区依托特色产业和资源优势，加快做强支柱产业，实现扬优发展；鼓励城区、景区提升产业发展水平和建设品位，实现提升发展。继续在政策、资金、项目、人才等方面加大支持力度，促进九江经济技术开发区在新起点上实现更快发展。推进共青城与德安、永修与南昌桑海、彭泽与湖口相向发展，推进九江县、湖口县快速融入主城区，推进星子由景区景点向旅游城市发展。支持修水、都昌、瑞昌向中等城市发展；支持武宁依托生态优势打造“养生”城市；支持共青城申报国家级开发区，打造新兴产业集聚区、转型发展先行区、和谐创业示范区。力争县县财政总收入过6亿，一半以上过10亿，4个以上过15亿。

　　促进旅游经济纵深发展。启动新一轮旅游发展总体规划修编，进一步完善各县（市、区）旅游发展总体规划和重点景区规划，设立旅游发展专项资金1000万元，打造以庐山为龙头的大旅游格局，加快由旅游资源大市向旅游经济强市转变。积极推进中信庐山西海国际生态休闲度假区、东林大佛佛教文化游览区、中华贤母园、世外桃源等重大项目建设步伐，开工建设大千世界青少年科普教育基地、中美关系研讨会常年会址、云居山国际禅修院、中华两岸旅游文化创意产业基地等项目，做好庐山西海天堂国际旅游度假中心、永修吴城古镇恢复等项目前期工作，创建一批精品景区，打造庐山人文圣山、庐山西海度假休闲、星子和武宁生态养生、浔阳城历史文化、共青城创业宜居、鄱阳湖候鸟天堂等六大旅游品牌。加大统一营销力度，形成山上山下、城里城外、市县融合的旅游整体营销格局。继续实施特定节假日观光游览景区向市民免费开放。进一步巩固日韩、港台等传统入境市场，大力拓展欧美、东南亚等新兴入境市场，确保全年旅游接待总人次、旅游总收入增长15%以上。

　　促进商贸经济提升发展。积极引进国内外知名商业连锁企业，推进特色商业街区、星级宾馆、高档写字楼、美食街区等功能设施建设，优化配置城区商业网点及配套设施，提升商贸综合服务能力。加快九江现代综合大市场、21世纪家居中心、华东建材市场升级改造、湖口钢铁贸易中心、共青城纺织服装工贸城、都昌珍珠交易市场、彭泽鄱阳湖棉花交易市场、德安陆路物流港等市场建设，支持有条件的县区培植特色物流市场。对接国内外知名物流企业，依托长江港口码头，新建集商业仓储、商品配送、货物中转、物流服务等功能为一体的大型商贸物流综合园区，力争港口货物吞吐量达4500万吨，集装箱16万标箱。继续开展“家电下乡”，推进“万村千乡市场工程”，进一步完善农村流通体系。

　　促进文化产业繁荣发展。制订完善文化产业发展规划，大力支持文化演艺精品创作，努力策划和打造一到两个全省乃至全国知名的文化活动品牌，创作一批文化演艺精品；加快推进瑞昌青铜文化主题公园、共青城动漫园等项目，建设一批文化产业主题公园；积极发展广播电影电视、新闻出版、报业等文化产业，培育一批文化产业龙头企业；支持引导鄱湖珍珠、龙韵琴行等企业做大做强，积极申报一批国家和省级文化产业示范基地；加快建设市博物馆、文化艺术中心等一批公共文化项目；加快引进和培育一批文化产业人才，打造一支高素质文化人才队伍。

　　（六）以提升发展质量为重点，推动改革创新再求新突破

　　坚持把改革创新作为调结构、促转变的重要环节，把节能降耗、环境保护和生态建设作为重要抓手，加快转变经济发展方式，不断提升经济发展质量和水平。

　　深化改革开放。统筹推进农村综合改革，基本完成农垦系统国有企业改革，扎实推进国有林场改革；分类推进事业单位改革；深入推进行政管理、文化体制、公共管理、医疗卫生、国资监管和投融资体制等改革；积极实施财政、户籍、收入分配等综合性改革。加大对非公经济的政策支持力度，拓展非公企业发展空间，推动非公经济全面加快发展。鼓励全民创业，积极引导外出创业务工人员回乡发展，大力支持有条件的地方建设返乡创业园区。进一步完善出口促进政策，优化出口产品结构，不断做大对外贸易规模，实现外贸出口26亿美元以上。鼓励有条件的企业到境外投资，推进对外工程承包和劳务合作。

　　推动科技创新。进一步创新人才工作机制，盘活用足现有人才资源，加大引才引智力度，培育和造就一批发展急需的领军型、创新型和技能型人才队伍。继续实施科技兴市、质量兴市、品牌兴市战略，大力推进科技创新“六个一”工程，不断提高科技成果转化率和贡献率。支持一批过百亿企业建成省级技术中心，一批规模企业建成市级研发机构，力争专利申请量800件以上。依托省级恒盛科技企业孵化器和省级服务外包基地两个平台，争创国家级高新技术创业中心，培育一批专、精、尖的科技型企业。制订完善相关政策措施，加快发展新兴产业，推进产业结构调整升级，全市新兴产业所占比重提升5-10个百分点。

　　加强生态建养。加大鄱阳湖生态保护和修河源头治理力度，强化水质监测，保护一湖清水。实施减排工程，严格环境执法，加快淘汰落后产能，削减排放总量。完善污水处理设施运行管理，继续推行“以创促治”、“以奖促治”等农村环保激励机制，确保城乡饮用水源安全和农村环境安全。进一步提升鄱阳湖采砂统一管理水平。加强水土保持综合治理，完成24万亩水土流失治理任务。实施资源再生利用工程，加强共生、伴生矿产资源综合开发，鼓励余热余压发电，提高资源利用效率。

　　（七）以保障和改善民生为重点，推动社会建设再出新成效

　　继续强化民生保障。把保障和改善民生作为政府工作的出发点和落脚点，进一步加大民生投入，全面完成各项民生工程目标任务。把保障性安居工程作为重大民生工程，围绕“3-5年在中心城区新建500万平方米、10万套保障性住房，解决40万人住房需求”的目标，继续加大投入，加强调度，加快推进，努力实现“只要在九江就业创业，就能在九江安居乐业”。扎实开展扶贫开发，设立专项资金1000万元，形成开发扶贫和救助扶贫并进、专项扶贫和政策扶贫并重、政府扶贫和社会扶贫并举的“大扶贫”格局。进一步健全以社会保险、社会救助、社会福利、社会慈善为重点的社会保障体系，不断提高农村“五保”对象和城镇“三无”人员供养水平。继续加大“三院”建设和管理力度，抓好新型农村社会养老保险和城镇居民社会养老保险工作，积极探索社会化养老的新途径。进一步完善就业服务体系，重点解决好高校毕业生、农村转移劳动力、城镇就业困难人员就业。进一步拓宽农村居民增收渠道，着力增加中等收入家庭的财产性收入，建立稳定的职工收入增长机制，将公务员津贴补贴和事业单位绩效工资按政策落实到位。

　　在完成省定民生工程任务的基础上，本着“群众有盼求、财力可保障、年内能完成”的原则，着力办好十件民生实事：一是新开工建设各类保障性住房8万套，竣工8.8万套（含续建），完成农村危房改造5000户。二是投入帮扶资金2.8亿元以上，实施395个扶贫开发重点村整村推进，完成移民扶贫搬迁4200人、避灾移民搬迁1万人。三是实施农村安全饮水工程，解决30万农村人口安全饮水问题。四是新扩城郊蔬菜基地6000亩，完成中心城区第三批3家农贸市场标准化升级改造，新增农超对接点50家。五是新建城市管网35公里，安装路灯500杆（盏），整治改造人行道1万平方米、边街小巷30条。六是投入9300万元，改造城区配电网和10万户智能电表改造，提升城区18万户居民用电的可靠性。完成难点难户的供水一户一表安装改造9000户。新增管道天然气用户2万户。七是免费救治晚期血吸虫病人1600例，免费检查血吸虫病60万人次。八是建设信息化乡镇50个、行政村500个。建设“阳光价格”网络信息平台，开展网上“晒价”惠民活动。建设市图书馆“自助图书管理系统”。九是办理法律援助案件2000件。十是完成1000公里农村公路改造。

　　加强和创新社会管理。健全信访责任制，完善群众诉求表达和维护机制，加大信访积案和各类社会矛盾纠纷的排查调处力度，妥善处理好人民内部矛盾。建立新闻舆论引导应对机制，加强网络文化建设与管理，依法促进信息网络健康有序发展。注重发挥群团组织在社会管理和公共服务中的桥梁纽带作用。进一步加强基层组织建设、城乡社区建设和平安边界建设。深入推进社区矫正工作，完善流浪儿童的救助机制，做好刑释解教人员安置帮教和弱势群体救助工作。健全公共安全监管机制，严格落实安全责任制和事故责任追究制，深入开展隐患排查和整改，保障道路交通、危化物品、森林防火、城市消防等重点领域安全。启动“六五”普法，推进依法治市。建立健全突发事件应急体系，完善防灾救灾减灾设施，不断提高风险防范和应急处置能力。健全社会治安打防体系，加强社会管理综合治理，发挥社会齐抓共管作用，严厉打击各类违法犯罪活动，切实维护国家安全和社会稳定。

　　促进社会事业协调发展。坚持教育优先发展，逐步提高教育投入比例，加大幼教设施建设力度，优化中小学校网点布局，加强学校学生安全管理。提升高等教育质量，促进职业教育市场导向性办学，推进城乡教育协调发展和各类教育均衡发展。加快卫生基础设施建设，提升公共卫生和基本医疗卫生服务能力。完善农村三级卫生服务网络和社区卫生服务体系，落实国家基本药物制度，提高新农合筹资水平，降低群众就医费用。深入开展全民健身运动，完善公共体育设施建设，举办第十一届全市运动会。加大监管力度，确保食品药品质量安全。完善价格监测预警机制，抓好猪肉、粮食等大宗消费品储备工作，保持物价基本稳定。规范客运市场秩序，突出公交优先发展战略，加快城区和城郊公交线路整合，打造畅通九江，实现便捷出行。加强人口与计划生育工作，不断健全利益导向机制，促进人口长期均衡发展。完善国防动员、国防教育体系，加强国防后备力量和人民防空建设。深入开展双拥共建活动，推进军民融合发展。大力支持驻浔解放军和武警部队建设，不断巩固和发展军政军民团结。统筹抓好统计、审计、海关、检验检疫、防震减灾、消防、盐业、邮政、通讯、气象、水文、地方志、档案、保密、编制、对台、外事侨务、民族宗教、无线电管理、工会、共青团、妇女儿童、老龄、残疾人、红十字和慈善等工作，推动各项社会事业全面协调发展。

　　（八）以提高行政效能为重点，推动政府自身建设再树新形象

　　面对新形势、新任务、新要求，我们必须坚持依法办事、为民做事、务实成事、团结共事、廉洁干事，全力打造“勤政为民、务实高效、团结奋进、清正廉洁”的政府。

　　依法行政履职能。坚持在市委的核心领导下，自觉接受市人大及其常委会的法律监督和市政协的民主监督，认真办理人大代表建议和政协提案，虚心听取和采纳民主党派、工商联、无党派人士的意见，主动接受新闻舆论监督和社会公众监督。严格执行民主集中制，建立重大工程项目和重大决策风险评估机制，推进科学决策、民主决策、依法决策。集中整治乱收费、乱摊派、乱罚款和吃拿卡要、不作为、乱作为等损害群众利益的不正之风，全面规范行政行为，真正做到权为民所用。

　　重心下移抓落实。蓝图变现实，关键在落实。实现既定的目标任务，我们必须眼睛朝下看，身子向下沉，重心往下移。坚持深入群众抓落实，始终保持密切联系群众的工作作风，经常深入一线调查研究，到边远的地方察民情，到困难的地方解民忧，到贫困的地方帮民富，扎扎实实把群众最现实、最直接、最紧迫的事情办好。坚持破解难题抓落实，对用地、用工、资金、能源等制约发展的各类难题，找准根源，敢于上手，寻求突破，做到不畏难、不避难，扎扎实实把难题破解好。坚持推动项目抓落实，树立“抓项目就是抓发展，抓项目就是抓落实”的理念，把责任落实到具体项目，把力量摆布到具体项目，把措施强化到具体项目，以项目建设的成效考量工作落实的成效。

　　创优服务提效能。按照打造高效政府的要求，全面加强机关效能建设，营造人人讲效能、处处抓效能、事事高效能的浓厚氛围。认真开展影响发展环境的干部作风突出问题集中整治活动，重点解决干部队伍中“庸”、“懒”、“浮”、“赌”、“贪”等问题。开展企业评机关活动，集中整治“两头热、中梗阻”现象，严格实行行政效能问责，确保政令畅通。进一步减少审批环节，缩短审批时限，优化审批流程，推进网上审批，完善服务承诺、首问负责、一次性告知和限时办结等制度，让群众一看就明白，一听就清楚，一次就办完，切实提高服务效率。

　　惩防并举保清廉。坚持“标本兼治、综合治理、惩防并举、注重预防”的方针，认真贯彻落实党风廉政责任制和廉洁自律的各项规定，强化廉政风险防控，建立健全教育、制度、监督并重的惩防体系。加强廉政教育，从思想上筑牢反腐倡廉防线，做到对组织负责、对人民负责、对家人负责、对自己负责。推进网上监察系统平台建设，强化对重大工程、重点领域、重要环节的监

　　管，严厉查处各类违法违纪案件。倡导勤俭节约，降低行政成本，

　　最大限度把资金用于促发展、保民生。

　　各位代表，势头良好，自当乘势而上；任务艰巨，更须全力赶超。让我们在市委的坚强领导下，进一步坚定信心，硬化措施，扎实工作，排难奋进，为九江争得应有地位、人民过上小康生活作出新的更大的贡献，以优异的成绩迎接党的十八大胜利召开！
